# Reversion of heme-dependent metronidazole resistance in *Clostridioides difficile*

**DOI:** 10.1128/spectrum.01787-25

**Published:** 2025-11-14

**Authors:** Lillia Louis, Firyal Ramzan, Dale D.O. Martin, Abiola O. Olaitan

**Affiliations:** 1 Department of Biology, University of Waterloo 8430, Waterloo, Ontario, Canada; Tainan Hospital Ministry of Health and Welfare, Tainan, Taiwan

**Keywords:** antimicrobial resistance, heme, metronidazole, *nim* genes, *Clostridium difficile*, 5-nitroimidazole reductase

## Abstract

Since the emergence of epidemic *Clostridioides difficile* in the early 2000s, reduced susceptibility to metronidazole (MTZ), once a frontline antibiotic for treating *C. difficile* infection, has emerged. Consequently, MTZ is no longer a guideline-recommended antibiotic in many countries. Heme and Nim proteins have been shown to co-mediate MTZ non-susceptibility by increasing the MTZ minimum inhibitory concentration (MIC). This study aimed to identify drugs that reverse heme-dependent MTZ non-susceptibility in *C. difficile*. Drugs known to interfere with heme were screened for their ability to inhibit MTZ non-susceptible *C. difficile*. Broth and agar dilution methods were used to assess the reversion of MTZ non-susceptibility in diverse clinical isolates, and checkerboard assay was used to determine drug-MTZ interactions. Gene expression analysis was performed using quantitative PCR and reporter assays. We uncovered artemisinin (ART) as an adjuvant capable of resensitizing heme-dependent MTZ non-susceptible *C. difficile*. ART reduced elevated MTZ MIC (1–8 µg/mL) in non-susceptible strains to the susceptible level (≤0.5 µg/mL) despite the presence of heme. Additionally, we demonstrated that ART reverses *nim*-mediated MTZ resistance in *Bacteroides fragilis*. The findings showed that ART significantly reduced intracellular hemin levels in *C. difficile*, even in the presence of exogenous hemin. This study identifies an adjuvant that repotentiates MTZ activity in non-susceptible *C. difficile*, where resistance is co-mediated by heme and the *nim* gene, offering a novel approach to restore MTZ susceptibility in anaerobes.

**IMPORTANCE:**

Metronidazole (MTZ) is a widely used antibiotic for treating anaerobic bacterial infections, including *Clostridioides difficile* infection. However, the increasing emergence of MTZ resistance among anaerobic pathogens poses a significant threat to its continued clinical effectiveness. Heme-dependent MTZ resistance, one of the mechanisms of resistance, has been associated with poorer treatment outcomes in patients. In this study, we identified artemisinin (ART) and demonstrated that it can reverse heme-dependent MTZ resistance in diverse clinical isolates of *C. difficile*. Furthermore, ART effectively reversed resistance mediated by the nim genes in both *C. difficile* and *Bacteroides fragilis*. These findings open a possible avenue for restoring the efficacy of MTZ against resistant anaerobic bacteria, particularly those with the Nim-mediated resistance mechanism.

## INTRODUCTION

*Clostridioides difficile* is a major cause of hospital-acquired infections. Recent analyses estimate the incidence of *C. difficile* infection (CDI) at 5.0 cases per 10,000 patient-days globally and between 4.90 and 5.35 cases per 10,000 patient-days in Canadian acute care hospitals (1, 2). Although antibiotics are the primary treatment for CDI, the options are largely limited to metronidazole (MTZ), vancomycin, and fidaxomicin (3). MTZ was once the preferred drug for mild to moderate CDI; however, its effectiveness has declined over time, leading to it no longer being recommended in North America and Europe (4, 5).

Non-susceptibility to MTZ, including both high-level resistance and reduced susceptibility, has recently emerged in *C. difficile*. One resistance mechanism involves the pCD-METRO plasmid, which confers MTZ resistance independently of heme (6). A second, more widespread mechanism involves heme-dependent resistance or reduced susceptibility to MTZ (7, 8) and is also commonly observed among epidemic ribotypes, such as ribotype 027 (9, 10). Studies have shown that heme plays a central role in conferring MTZ non-susceptibility in *C. difficile* (7–9). In our recent study, 41% of a global collection of clinical *C. difficile* isolates exhibited heme-dependent MTZ non-susceptibility, with a ≥4-fold increase in minimum inhibitory concentration (MIC) in the presence of heme compared to its absence (9). This heme-dependent phenotype is mediated mostly by NimB (5-nitroimidazole reductase) in *C. difficile*, which uses heme as a co-factor to reduce MTZ to an inactive amine end product (9). Although the European Committee on Antimicrobial Susceptibility Testing (EUCAST) resistance breakpoint, based on epidemiological cut-off values, is >2 µg/mL, the heme-dependent MTZ non-susceptibility phenotype is defined by an MIC of ≥1 µg/mL and a ≥4-fold increase in MIC in the presence of heme compared to its absence. Reduced MTZ susceptibility has been shown to correlate with treatment failures and was identified as an independent predictor of initial clinical failure in patients being treated with an MTZ-based regimen (11).

In approximately 90% of cases, heme-dependent MTZ non-susceptibility arises from a unique mutation in the *nimB* promoter (P*nimB*^G^), which leads to constitutive expression of *nimB*, in contrast to susceptible strains that carry the wild-type promoter (P*nimB*^T^) (9). As a result, MTZ non-susceptible strains exhibit elevated MTZ MICs in the presence of heme but low MICs in its absence, whereas susceptible strains maintain low MICs regardless of heme availability (9). A recent analysis of *C. difficile* genomes from public databases found the P*nimB*^G^ promoter mutation in 5,199 out of 26,557 (19.6%) isolates, indicating the global spread of this resistance phenotype (10).

Resistance to the current antimicrobials used to treat CDI, namely MTZ, vancomycin, and fidaxomicin, is increasing and has been linked to treatment failure in patients (11–14). The increasing incidence of antimicrobial resistance, coupled with the lack of new antibiotics, poses a significant threat to the effective management of bacterial infections. One strategy to address this problem involves combining antibiotics with adjuvants to resensitize resistant bacteria (15, 16). The objective of this study was therefore to screen and identify drugs that can reverse heme-dependent MTZ non-susceptibility in *C. difficile* by targeting heme, given its critical role in MTZ resistance. Here, we identified artemisinin (ART) as capable of reversing heme-dependent MTZ non-susceptibility. We demonstrated that ART directly restores the *in vitro* efficacy of MTZ in diverse resistant clinical *C. difficile* strains, reducing their heme-dependent elevated MICs to levels similar to those of susceptible strains. Our findings show that ART resensitizes *C. difficile* to the toxic effects of MTZ, as demonstrated by the reactivation of the transcriptional signatures of MTZ-response genes initially suppressed by heme.

## RESULTS

### Screening identified inhibitor of heme-dependent MTZ non-susceptibility

**Fig 1:**
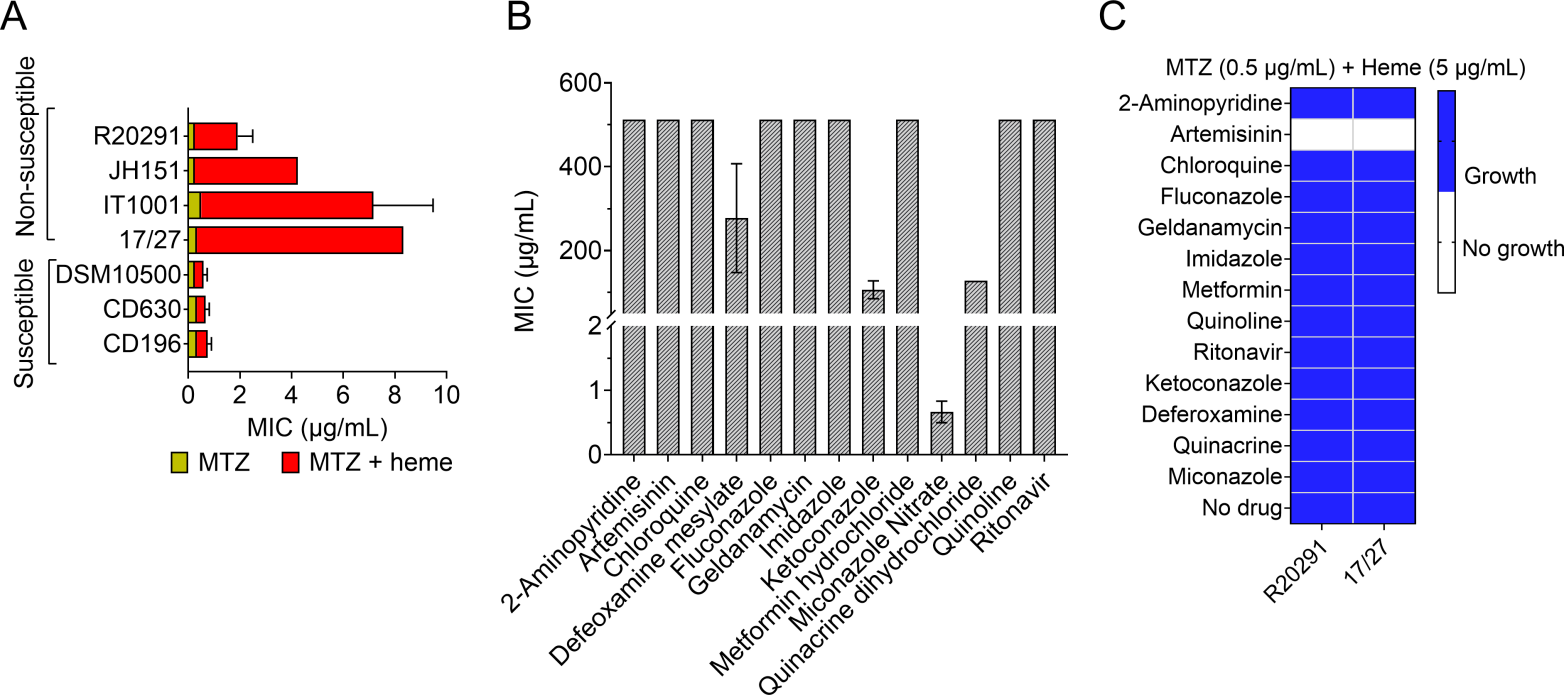
(**A**) MTZ MIC reevaluation in select *C. difficile* isolates. In the absence of heme, all seven tested isolates displayed susceptibility to MTZ (≤0.5 µg/mL). However, only strains R20291, JH151, IT1001, and 17/27 exhibited heme-dependent MTZ non-susceptibility, showing at least a fourfold increase in MICs (2–8 µg/mL) in the presence of heme. (**B**) Screening for antibacterial effect of curated drugs. Using R20291, all drugs except miconazole nitrate exhibited MICs ≥64 µg/mL, indicating a lack of antibacterial activity against *C. difficile*. The maximum concentration tested was 512 µg/mL. MICs from (**A**) and (**B**) are shown as the mean ± SEM from three biological replicates. (**C**) Drug screening identifies ART as an inhibitor of heme-dependent MTZ-non-susceptible *C. difficile*. Among all drugs tested in the presence of 0.5 µg/mL MTZ and 5 µg/mL heme, only ART inhibited the growth of the two MTZ non-susceptible *C. difficile* strains (R20291 and 17/27). Blue indicates growth, while white indicates no growth. Screening was performed in two biological replicates.

We first reassessed the MIC of MTZ with and without heme, for seven *C. difficile* isolates. In the absence of heme, all isolates tested displayed low MTZ MICs of 0.25–0.5 µg/mL (Fig. 1A). However, in the presence of heme, four of these isolates (R20291, JH151, IT1001, and 17/27) exhibited elevated MTZ MIC values, ranging from 2 to 8 µg/mL (Fig. 1A), corroborating previous reports (7, 9).

To identify drugs that could reverse heme-dependent MTZ non-susceptibility, we conducted an extensive bibliographical search to collate drugs known to interact with heme, resulting in 13 candidate compounds (Table S1). We then assessed the MICs of these compounds against a representative MTZ non-susceptible *C. difficile* strain, R20291. The MICs ranged between 0.5 and 512 µg/mL (Fig. 1B). Only miconazole showed a low MIC of 0.5–1  µg/mL, whereas all other compounds had MICs ≥ 64 µg/mL, indicating that they are non-antibiotic against *C. difficile* and could serve as potential adjuvant candidates (15).

Screening was conducted using MTZ at 0.5 µg/mL with heme (5 µg/mL) to identify compounds capable of reversing heme-dependent MTZ non-susceptibility, since susceptible strains exhibited MTZ MICs ≤ 0.5 µg/mL regardless of heme presence. Drugs were screened at their respective 1/4× MIC in the presence of the specified MTZ and heme concentrations. We utilized two MTZ non-susceptible strains for the screening: R20291 (MIC 2 µg/mL) and 17/27 (MIC 8 µg/mL), representing distinct genotypes. Strain R20291 carried the P*nimB*^G^ promoter, whereas strain 17/27 lacked the SNP. In both strains, MTZ resistance has been shown to be modulated by heme and *nimB* (9). We found that out of 13 drugs, only ART prevented the growth of both strains in 0.5 µg/mL MTZ (Fig. 1C). The MTZ-susceptible strain CD196, used as a negative control, remained susceptible (data not shown). As an additional control, we tested whether ART could reverse vancomycin resistance, a non-heme-dependent mechanism, in resistant strains (MT14700, 490054, and JH7). The results showed that ART did not reverse vancomycin resistance in these strains (Fig.S1). Furthermore, we tested the ART analog artesunate and the non-ART analog chloroquine. Artesunate exhibited a similar ability to ART in reversing MTZ non-susceptibility, whereas chloroquine showed no such effect (Fig. S2). These results indicated that ART was specifically capable of preventing the growth of heme-dependent MTZ non-susceptible *C. difficile* strains at the MTZ MIC to which they were initially resistant.

### ART reversed heme-dependent MTZ non-susceptibility in diverse clinical isolates

**Fig 2:**
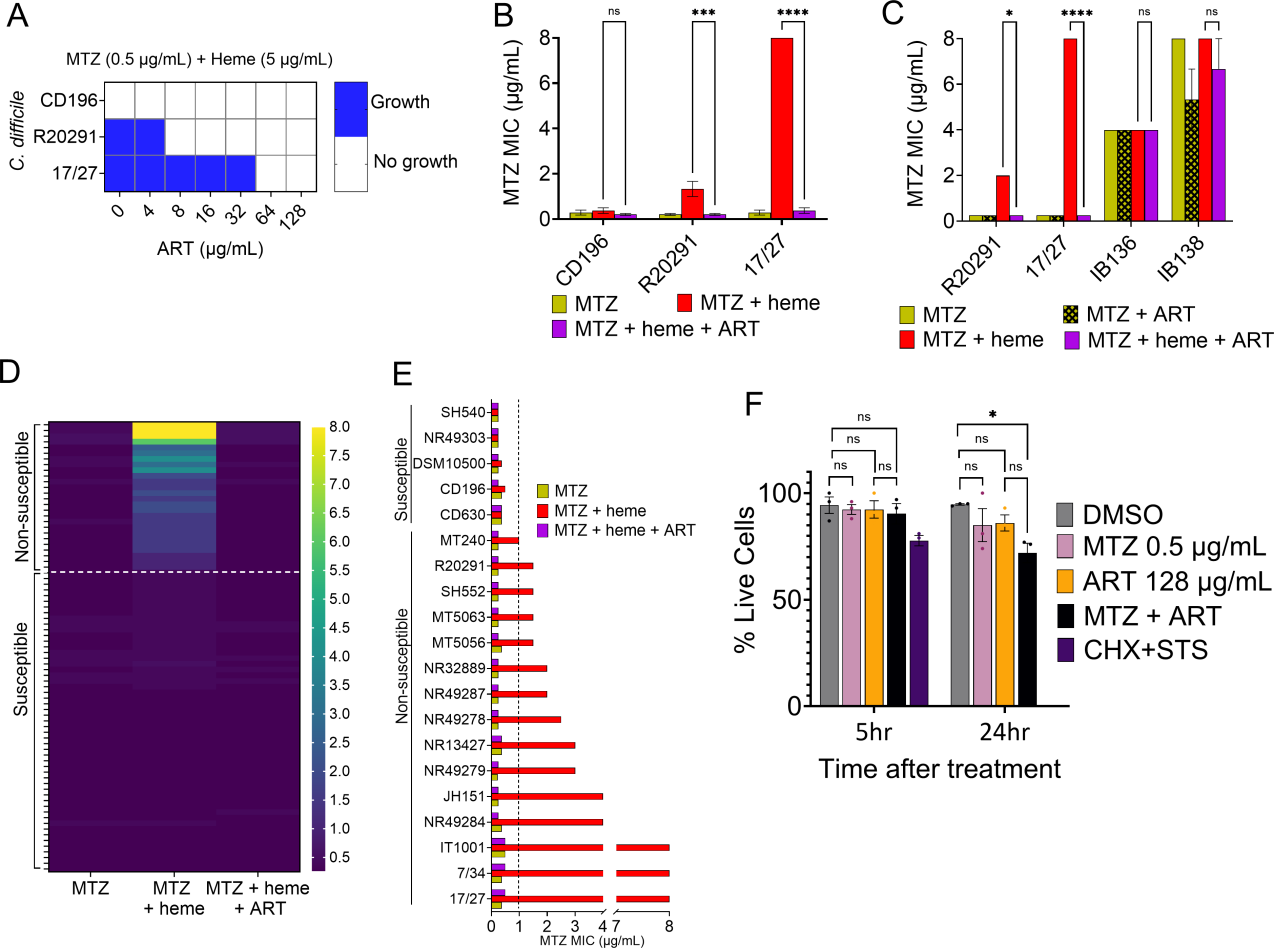
(**A**) Evaluation of effective concentrations of ART capable of reversing heme-dependent MTZ non-susceptibility. MTZ non-susceptible R20291 and 17/27 were resensitized to MTZ by ART at 8 and 64 µg/mL, respectively. The MTZ-susceptible control strain CD196 was inhibited by MTZ, without ART supplementation. Blue indicates growth, while white indicates no growth. (**B**) Range of MTZ MIC reduction by ART. ART restored MTZ susceptibility in R20291 and 17/27 to levels comparable to those without heme or the susceptible CD196 control (0.125–0.5 µg/mL). (**C**) ART reversed MTZ resistance mediated by the heme-dependent mechanism (strains R20291 and 17/27), but not resistance mediated by the heme-independent pCD-METRO plasmid (strains IB136 and IB138). Data from (**B**) and (**C**) represent the mean ± SEM from three biological replicates. ns: not significant; *: *P* ≤ 0.05; ***: *P* ≤ 0.001; and ****: *P* ≤ 0.0001 (two-way ANOVA with Holm-Šídák multiple comparisons test). (**D**) Heatmap showing the reversion of MTZ non-susceptibility by ART across a broad range of *C. difficile* clinical isolates. ART reversed the elevated MTZ MICs (1–8 µg/mL) in all 26 non-susceptible isolates to levels comparable to those observed without heme or in all susceptible isolates (0.25–0.5 µg/mL). This reversion was also observed in MTZ-non-susceptible strains lacking the P*nimB*^G^ mutation (17/27, 7/34, IT1001, IT1002, MT5056, and MT5063). MICs are shown as the mean from two biological replicates. (**E**) A subset of MTZ-susceptible and non-susceptible isolates from (**D**) showing the reversion of MTZ resistance. The dashed lines in (**D**) and (**E**) indicate our defined MTZ non-susceptibility breakpoint of 1 µg/mL for heme-dependent MTZ non-susceptible isolates. (**F**) Trypan blue exclusion assay of MTZ, ART, and their combination on HEK293T cells. Relative to the vehicle (DMSO) control, all treatments maintained ≥90% cell viability after 5 h. After 24 h, MTZ and ART remained non-toxic individually, while their combination reduced viability to ~76%, suggesting mild cytotoxicity. Cycloheximide (CHX; 10 μg/mL) and staurosporine (STS; 1 μM) combination served as a positive control, causing complete cell death after 24 h. Data represent the mean ± SEM from three biological replicates. ns: not significant; *: *P* ≤ 0.05 (one-way ANOVA with Tukey’s multiple comparisons test).

We first evaluated the range of ART concentrations effective in repotentiating MTZ against the non-susceptible *C. difficile* strains (R20291 and 17/27). ART concentrations ranging from 0 to 128 µg/mL were tested in the presence of 0.5 µg/mL MTZ with 5 µg/mL heme. We observed that ART at 8 and 64 µg/mL inhibited the growth of R20291 and 17/27, respectively, at the tested MTZ concentration of 0.5 µg/mL (Fig. 2A). Our results also revealed that neither ART alone nor its combination with heme inhibited the growth of *C. difficile*, indicating that ART itself indeed repotentiates MTZ activity in these strains (Fig. S3).

Next, we evaluated the new susceptible MTZ MIC at which ART reduced the initial MIC of these non-susceptible strains. Since the highest overall effective ART concentration was 64 µg/mL for strain 17/27, we used double this concentration, 128 µg/mL. The results demonstrated that ART at 128 µg/mL reduced the MTZ MIC for R20291 from 2 to 0.125–0.25 µg/mL, and for 17/27 from 8 to 0.125–0.5 µg/mL (Fig. 2B). These new MICs were comparable to those of the susceptible CD196 strain (MIC of 0.125–0.5 µg/mL in the presence or absence of heme; Fig. 2B). Then, using the checkerboard assay, we demonstrated that the combination of MTZ and ART, in the presence of heme, exhibited a synergistic effect against the non-susceptible R20291 and 17/27 (fractional inhibitory concentration index [FICI] ≤ 0.375, Table 1).

**TABLE 1:** Fractional inhibitory concentration index for ART and MTZ interaction in MTZ non-susceptible *C. difficile*

	Individual MIC^*a*^		Combination MIC^*b*^		FIC			
	MTZ	ART	MTZ	ART	MTZ	ART	FICI	Interpretation
R20291	2	512	0.125–0.25	32–128	0.0625–0.125	0.0625–0.125	0.125–0.375	Synergistic
17/27	8	512	0.25–0.5	32–64	0.03125–0.0625	0.0625–0.125	0.09375–0.125	synergistic

^*a*^ Individual MIC refers to the MIC of metronidazole (MTZ) or artemisinin (ART) when tested alone.

^*b*^ Combination MIC refers to the MIC of MTZ or ART when tested in combination during the assay. FIC, Fractional inhibitory concentration; FICI, fractional inhibitory concentration index.

Another mechanism of MTZ resistance involves the plasmid pCD-METRO, which confers resistance independently of heme (6). Accordingly, we anticipated that ART would not be effective in reversing this form of resistance. As expected, while ART successfully reversed MTZ non-susceptibility in the R20291 and 17/27 strains, it had no effect on the pCD-METRO-carrying isolates IB136 and IB138, where their MICs remained unchanged (4–8 µg/mL) regardless of treatment with ART (Fig. 2C). These findings demonstrated that ART specifically targets heme-dependent MTZ non-susceptibility.

We next assessed the efficacy of ART in reversing MTZ non-susceptibility in various *C. difficile* isolates. We analyzed 79 clinical isolates from diverse ribotypes and geographical locations. Among these, 26 isolates were MTZ non-susceptible, with MICs ranging from 1 to 8 µg/mL in the presence of heme and ≤0.25–0.5 µg/mL in the absence of heme (Fig. 2D; Table S2). The remaining 53 isolates remained susceptible, with MTZ MICs of ≤0.25–0.5 µg/mL regardless of heme presence. The results showed that the addition of ART (128 µg/mL) to the MTZ with heme condition effectively reduced the MICs in all MTZ non-susceptible strains, including MTZ non-susceptible isolates lacking the P*nimB*^G^, to levels comparable to those of the susceptible strains or the MICs of MTZ without heme (Fig. 2D). Specifically, ART reversed MTZ non-susceptibility in all 26 non-susceptible isolates, lowering the MICs from 1 to 8 µg/mL to ≤0.25–0.5 µg/mL, thus eliminating the ≥4-fold increase in MTZ MIC induced by heme in all strains (Fig. 2E).

We assessed the cytotoxicity of MTZ, ART, and their combination in a mammalian cell line (human embryonic kidney cells, HEK293T). ART and MTZ, alone or in combination, showed no acute cytotoxicity after 5 h of treatment compared with vehicle (DMSO)-treated cells. After 24 h, ART and MTZ were non-toxic, while their combination displayed mild toxicity (Fig. 2F). Further, we tested whether the addition of ART would lead to the emergence of MTZ resistance. The results showed no increase in MTZ MIC over 16 serial passages under subinhibitory concentrations of ART and MTZ (Fig. S4). Taken together, these results showed that ART worked synergistically with MTZ and was effective in reversing the heme-dependent MTZ resistance in diverse *C. difficile* strains, including those possessing or lacking the P*nim*B^G^ SNP, restoring them to the level of susceptible strains.

### ART inhibited *nimB***-**mediated MTZ non-susceptibility and reduced intracellular hemin

**Fig 3:**
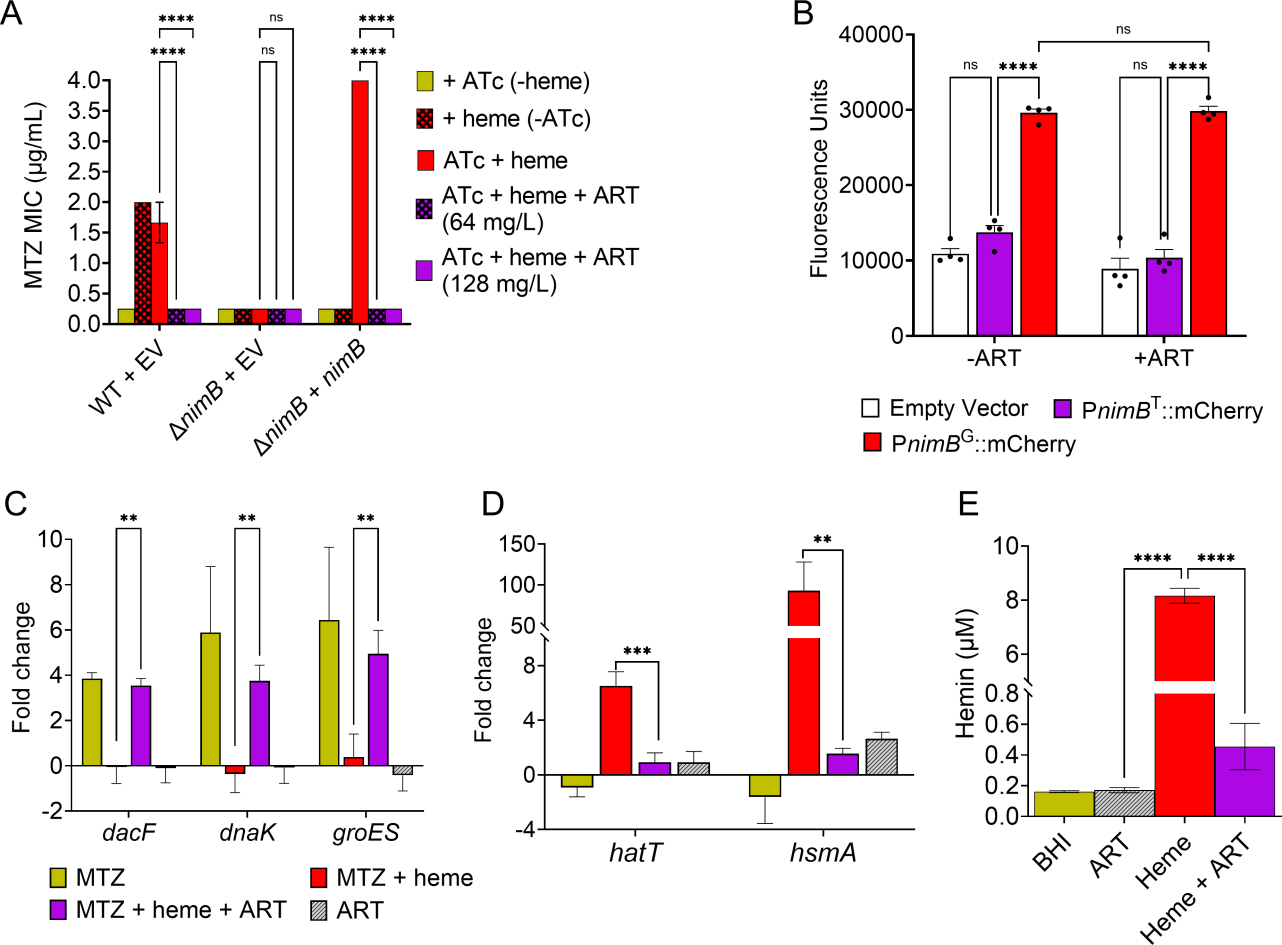
(**A**) ART reverses MTZ non-susceptibility mediated by *nimB*. In the R20291 WT strain, *nimB* mediates heme-dependent MTZ non-susceptibility. The Δ*nimB* mutant was susceptible, and complementation (Δ*nimB + nimB*) restored MTZ non-susceptibility. ART at 64 or 128 µg/mL reversed *nimB*-mediated MTZ non-susceptibility in both the WT and complemented strains. MIC values are the mean ± SEM from three biological replicates. ns: not significant; ****: *P* ≤ 0.0001 (two-way ANOVA with Holm-Šídák multiple comparisons test). (**B**) Evaluation of ART on *nimB* promoter activity. A transcriptional mCherryOpt reporter driven by the mutant *nimB* promoter (P*nimB*^G^::mCherry) showed significantly increased promoter activity in R20291 in the presence of MTZ and heme, whereas the WT promoter (P*nimB*^T^::mCherry) and EV control did not. ART treatment did not suppress the increased promoter activity of P*nimB*^G^::mCherry. Data represent the mean ± SEM from four biological replicates. ns: not significant; ****: *P* ≤ 0.0001 (two-way ANOVA with Tukey’s multiple comparisons test). (**C**) In R20291, the MTZ-responsive genes (*dacF*, *dnaK*, and *groES*) were activated under MTZ-susceptible conditions (MTZ only) but remained uninduced under MTZ-resistant conditions (MTZ + heme). Supplementation with ART in the resistant condition reactivated these genes, similar to susceptible conditions. Data represent the mean ± SEM from four biological replicates. **: *P* ≤ 0.01 (two-way ANOVA with Holm-Šídák multiple comparisons test). (**D**) The heme-responsive genes *hatT* and *hsmA* were activated in the presence of heme (MTZ + heme) but were repressed in the presence of ART. Gene expression levels are shown relative to the DMSO control. Data represent the mean ± SEM from four biological replicates. (**E**) Quantification of intracellular hemin levels. Hemin supplementation markedly increased intracellular hemin in *C. difficile* R20291, while co-treatment with ART significantly reduced hemin intracellularly compared to hemin treatment alone. DMSO and ART-only treatments showed only baseline levels. Data are presented as the mean ± SEM from three biological replicates. Statistical analyses for (**D**) and (**E**): **: *P* ≤ 0.01; ***: *P* ≤ 0.001; ****: *P* ≤ 0.0001 (one-way ANOVA with Holm-Šídák multiple comparisons test).

A previous study has shown that *nimB* mediates heme-dependent MTZ non-susceptibility in *C. difficile* (9). To directly link ART ability to reverse heme-dependent MTZ resistance to *nimB*-mediated resistance, we used *C. difficile* R20291 lacking *nimB* (Δ*nimB*, MTZ-susceptible) and a complemented strain expressing *nimB* (Δ*nimB + nimB*, MTZ non-susceptible). As expected, the Δ*nimB* EV (empty vector [EV]) was susceptible to MTZ (MIC 0.25 µg/mL) (Fig. 3A). The complemented strain (Δ*nimB + nimB*) with activated *nimB* (ATc induction and heme) displayed increased MTZ MIC (4 µg/mL), similar to the non-susceptible WT EV with an intact *nimB* gene (1–2 µg/mL; Fig. 3A). The complemented strain remained susceptible to MTZ (MIC 0.25 µg/mL) when *nimB* expression was not activated (absence of ATc) or when heme was absent. Notably, the addition of ART restored MTZ susceptibility in the Δ*nimB + nimB* complemented strain, reducing the MIC from 4 to ≤0.25 µg/mL, similar to the MTZ-susceptible Δ*nimB* or the non-*nimB* activated complemented strain (Fig. 3A). These findings demonstrated that ART effectively reversed MTZ non-susceptibility conferred by *nimB* in *C. difficile*.

It has been demonstrated that MTZ non-susceptibility co-mediated by heme and *nimB* was majorly caused by a unique mutation (T to G) in the *nimB* promoter of resistant isolates (P*nimB*^G^), leading to their increased promoter activity and constitutive *nimB* expression compared to susceptible isolates (9, 10). We investigated whether ART reversed MTZ resistance by reducing the *nimB* promoter activity by comparing the promoter activity of P*nimB*^G^ and P*nimB*^T^ in the presence of ART. As expected, the results showed that the reporter carrying P*nimB*^T^ displayed no promoter activity and was comparable to the EV, irrespective of ART presence (Fig. 3B). On the other hand, the reporter with P*nimB*^G^ showed significantly elevated promoter activity relative to P*nimB*^T^. However, the addition of ART did not reduce P*nimB*^G^ elevated promoter activity (Fig. 3B).

We next investigated how ART influenced the transcription of MTZ-responsive genes in the non-susceptible strain R20291. In the presence of MTZ alone (susceptible condition), MTZ-responsive genes *dacF*, *dnaK*, and *groES* were induced. However, when heme was included (non-susceptible condition), their activation was suppressed, suggesting that heme mitigates MTZ toxicity. The addition of ART in the non-susceptible condition (MTZ and heme) restored the induction of these genes to levels similar to those the susceptible condition (Fig. 3C), indicating that ART resensitized the bacteria to MTZ’s toxic effects. ART alone did not markedly influence these genes.

To explore ART’s impact on heme-mediated resistance, we used the heme-responsive genes *hatT* and *hsmA*, which are known to be upregulated in the presence of heme in *C. difficile*, as surrogate markers of intracellular heme levels (17, 18). Under MTZ with heme condition, heme activated the expression of *hatT* and *hsmA*, while the addition of ART suppressed this activation (Fig. 3D), suggesting that ART reduced intracellular heme availability. We further quantified intracellular hemin levels in *C. difficile* using a hemin assay kit. No substantial hemin was detected in cells grown in BHI or treated with ART alone. In contrast, hemin supplementation led to a pronounced increase in intracellular hemin (8.2  µM), which was significantly reduced by co-treatment with ART (0.5  µM) (Fig. 3E). This finding aligns with the gene expression data (Fig. 3D), suggesting that ART reduces intracellular hemin accumulation in *C. difficile*. Taken together, these findings showed that ART reduced intracellular hemin levels, reversed *nimB*-mediated MTZ non-susceptibility, and resensitized *C. difficile* to MTZ toxicity.

### ART reversed MTZ non-susceptibility mediated by *nim* gene in *B. fragilis*

**Fig 4:**
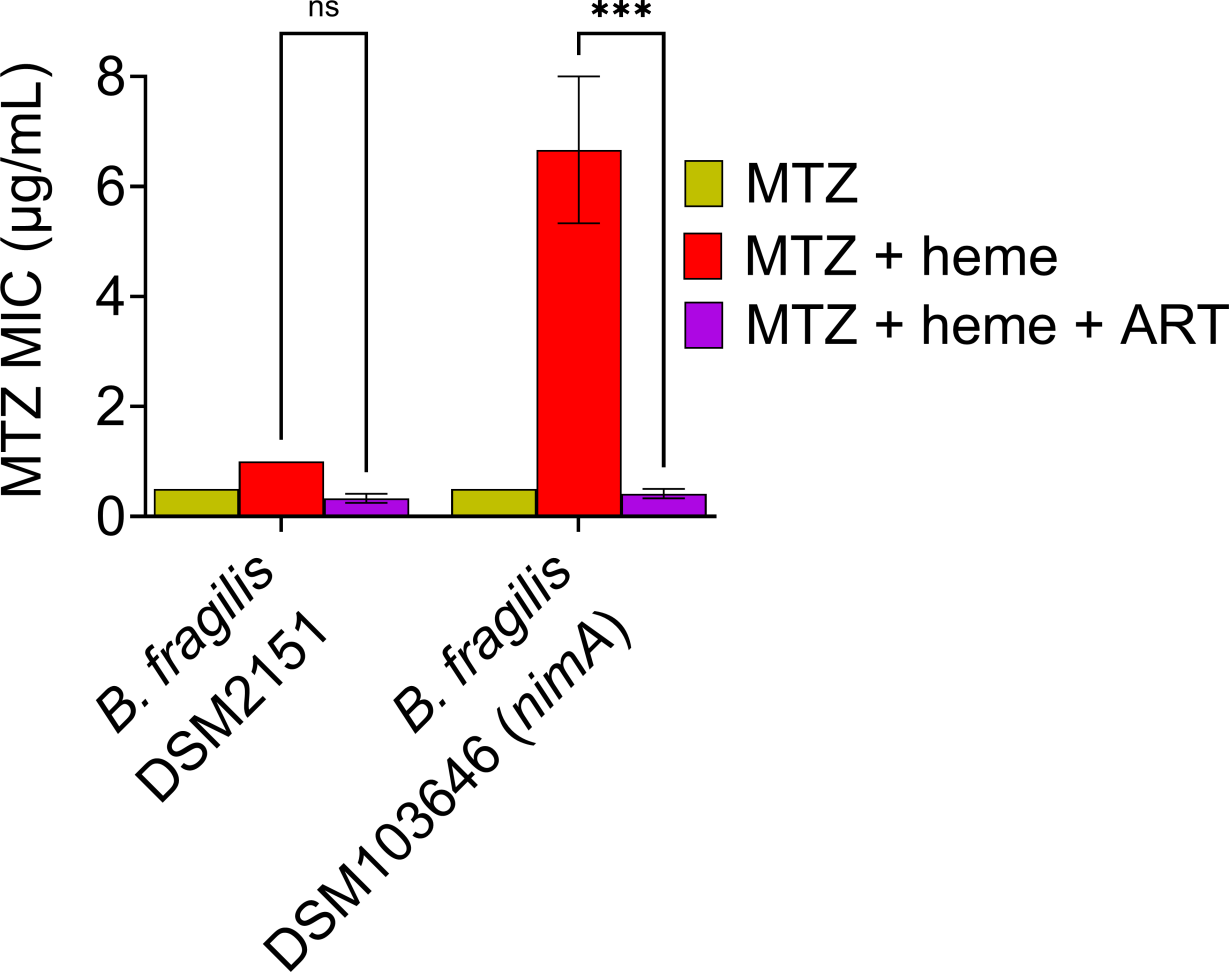
ART restores MTZ susceptibility in *B. fragilis* by reversing *nimA*-mediated resistance. In *B. fragilis*, the *nimA*-positive strain (DSM103646) exhibited heme-dependent MTZ non-susceptibility, which was reversed by ART treatment, restoring susceptibility to levels comparable to the *nimA*-negative MTZ-susceptible strain (DSM2151). MICs are shown as the mean ± SEM from three biological replicates. ns: not significant; ***: *P* ≤ 0.001 (two-way ANOVA with Holm-Šídák multiple comparisons test).

Similar to its role in *C. difficile*, *nim* has been widely recognized for mediating MTZ non-susceptibility in *Bacteroides* spp. (19), with heme also contributing to the resistance phenotype (9, 20). To investigate whether ART could reverse MTZ non-susceptibility in *B. fragilis* co-mediated by *nim* and heme, we utilized two strains: MTZ-susceptible *B. fragilis* DSM2151, which lacks the *nimA* gene, and MTZ-resistant *B. fragilis* DSM103646, which harbors *nimA*. Both strains were susceptible to MTZ in the absence of heme, with an MTZ MIC of 0.5 µg/mL (Fig. 4). However, only *B. fragilis* DSM103646, which carries *nimA*, exhibited resistance in the presence of heme, with an MIC of 8 µg/mL (Fig. 4). The addition of ART reversed the MTZ non-susceptibility of *B. fragilis* DSM103646, reducing the MIC from 8 to ≤0.5 µg/mL (Fig. 4). *B. fragilis* growth remained unaffected in the presence of ART (128 µg/mL) supplemented with heme (Fig. S5). These findings demonstrated that ART could restore MTZ non-susceptibility in other anaerobes, particularly in *B. fragilis*, where resistance is similarly mediated by *nim* and heme.

## DISCUSSION

The epidemiology of *C. difficile* has shifted significantly since the emergence of epidemic hypervirulent strains in the early 2000s, coinciding with a decline in the effectiveness of MTZ in treating CDI. For example, treatment failure rates in Quebec, Canada, increased from 9.6% during 1991–2002 to 25.7% in 2003–2004 (21). Reduced susceptibility to MTZ has been linked to reduced clinical response rates in patients (11).

Given that heme plays a central role in MTZ non-susceptibility in *C. difficile*, a process also co-mediated by Nim, a hemoprotein (7–9), we hypothesized that targeting heme-mediated activity could be a viable strategy to reverse this phenotype. In this study, we found that ART reversed the elevated heme-dependent MTZ MIC in non-susceptible *C. difficile*. ART acted synergistically with MTZ to restore susceptibility. ART is a well-known antimalarial drug that has also been extensively studied for other clinical indications, including anticancer, anti-inflammatory, and antiviral therapies, highlighting its broad therapeutic potential (22–25).

At least 12 Nim homologs (NimA–NimL) have been identified in clinically relevant anaerobic pathogens, including *Bacteroides* spp., with some being heme-dependent (9, 19, 20, 26–28). Several of these homologs are associated with MTZ resistance across multiple infection types (19, 26, 29). Our study demonstrated that ART effectively reversed MTZ non-susceptibility co-mediated by heme and *nim*, including in *B. fragilis*, where resistance is mediated by a similar mechanism, thus highlighting its potential therapeutic significance beyond *C. difficile*. At 128  µg/mL, ART, alone or with heme, showed no antibacterial activity against *C. difficile*, supporting its role as an adjuvant rather than a direct antimicrobial. The ART and heme combination similarly did not inhibit the growth of *B. fragilis*, despite its heme requirement; residual intracellular heme likely supported growth but was insufficient to confer MTZ resistance. However, at 256  µg/mL, ART inhibited *B. fragilis* growth, likely due to greater heme depletion (data not shown). These findings indicate that the death of MTZ-resistant bacteria results from ART-mediated resensitization to MTZ rather than direct growth inhibition via heme deprivation. Further, ART is known to undergo heme-mediated activation, generating reactive radicals that contribute to parasite death (30). In our study, *C. difficile* and *B. fragilis* grew in the presence of ART supplemented with heme, indicating that bacterial death was not due to heme-activated ART; instead, ART resensitized MTZ in heme-dependent resistant strains.

Although NimB-mediated MTZ resistance is driven by constitutive expression of *nimB* (9), our findings ruled out repression of *nimB* as the mechanism of ART activity. This mechanism may explain why ART reverses heme-dependent MTZ non-susceptibility, regardless of whether resistance is mediated by P*nimB*^G^. In non-susceptible strains lacking this mutation, MTZ resistance appears to be mediated, at least in part, by *nimB* (9), likely influenced by an unidentified genetic factor that also modulates *nimB*. We further found that ART significantly reduces intracellular hemin levels in *C. difficile*, even in the presence of exogenous hemin, indicating that it effectively limits heme bioavailability. This restriction likely underlies how ART restores MTZ susceptibility in heme-dependent resistant strains, as NimB requires heme as a cofactor (9). Indeed, ART is known to chemically alkylate free heme, forming covalent heme-ART adducts in *Plasmodium* sp (31–34), suggesting that a similar mechanism may operate here, although further investigation is needed to confirm this possibility.

Our *in vitro* studies show that the effective ART concentration required to reverse MTZ non-susceptibility in *C. difficile* is 64 µg/ml, which is far higher than its bioavailable plasma concentration (0.45 to 0.587 µg/ml) in terms of its pharmacokinetic-pharmacodynamic (PK-PD) (35–37). However, the colonic or fecal concentrations of ART remain unknown (35–37). Previous studies have shown that ART and its derivative, dihydroartemisinin, can ameliorate inflammatory bowel disease, a colonic disease with pathophysiological similarities to CDI, and help restore disrupted gut microbiota (38, 39). These findings suggest that achieving a therapeutically relevant colonic PK-PD profile for ART is feasible, potentially through targeted colonic delivery strategies to enhance *in vivo* bioavailability (40). Although the ART and MTZ combination showed mild cytotoxicity on mammalian cell line, our study provides the first *in vitro* proof of concept that MTZ non-susceptibility, mediated by heme and *nim*, can be reversed in bacterial pathogens such as *C. difficile*. Our findings could guide future efforts to develop new MTZ analogs that are non-toxic and effective against Nim-mediated resistance. This resistance mechanism is particularly prevalent among epidemic hypervirulent *C. difficile* lineages associated with severe CDI, for which MTZ is currently not recommended (9, 10). This study has some limitations. First, it does not include a mouse model to assess whether ART can improve outcomes in mice infected with MTZ-resistant *C. difficile*. Second, the mechanistic understanding of how ART reverses heme-mediated resistance is limited. Further studies are needed to evaluate the *in vivo* efficacy and safety of ART in the context of MTZ resistance and to elucidate the precise mechanistic basis of its action on Nim in reversing heme-mediated MTZ non-susceptibility.

In conclusion, this study identified ART as an MTZ adjuvant that restores MTZ susceptibility in resistant *C. difficile* and *B. fragilis*, where resistance is co-mediated by heme and *nim*. These findings present a promising strategy for overcoming MTZ resistance in anaerobic pathogens.

## MATERIALS AND METHODS

### Bacterial strains

*C. difficile* strains R20291 and 17/27 were used as representative MTZ-resistant strains, and CD196 as the susceptible strain. Additional clinical isolates, some of which have been previously reported (9), included those from the Texas Medical Center and Biodefense and Emerging Infections (BEI) Resources. The MTZ-resistant *C. difficile* strains IT1001 and IT1002 from Italy, as well as the pCD-METRO-bearing strains IB136 and IB138 used in this study, were previously reported (6, 41). *Bacteroides fragilis* DSM103646 (MTZ-resistant, *nimA*-positive) and DSM2151 (MTZ-susceptible, *nimA*-negative) were obtained from Leibniz Institute DSMZ. All isolates were cultured in pre-reduced brain heart infusion (BHI) broth or agar, with *B. fragilis* maintained on BHI agar supplemented with 5 µg/mL hemin and incubated anaerobically at 37°C in a Whitley A35 anaerobic workstation containing 10% H_2_, 5% CO_2_, and 85% N_2_ (Don Whitley Scientific).

### Susceptibility tests and drug screening

Broth-based MTZ MICs were determined using doubling dilutions of MTZ (0.25–16 µg/mL; Sigma Aldrich, Cat. No. M3761) with 5 µg/mL hemin (Toronto Research Chemicals, Cat. No. TRC-H245648). The 5 µg/mL hemin concentration used in this study is similar to the standard recommended by EUCAST for susceptibility testing and to that used in media such as Wilkins-Chalgren agar. A 0.5% overnight *C. difficile* culture (100 µL) was used for broth-based MIC assays. Agar-based MTZ MICs followed the same dilution range with hemin, pre-reduced for at least 1 h before inoculation with 2 µL of overnight culture. For *B. fragilis*, we used overnight cultures grown in hemin-free BHI broth (to prevent hemin carryover) from cultures on hemin-supplemented BHI agar.

Drug MICs were evaluated using broth dilution with doubling dilutions (0.5–512 µg/mL) of test drugs in BHI in 96-well microplates (100 µL). The pre-reduced plate was inoculated with 100 µL of a 10% overnight R20291 culture and incubated for 24 h. All drugs were sourced from Toronto Research Chemicals (Canada), Sigma Aldrich, Cayman Chemical, and Chem-Impex (Table S1) and dissolved in DMSO to a stock concentration of 10,000 or 20,000 µg/mL for ART. MTZ inhibition by drugs was screened at 1/4× MIC of drugs in MTZ (0.5 µg/mL) plus hemin (5 µg/mL) in 96-well microplates and inoculated with overnight cultures diluted to 0.5%, covered with aluminum foil, and incubated anaerobically for 24 h. Bacterial growth was assessed based on visible bacterial growth on agar plates or turbidity in broth cultures.

For assays with Δ*nimB* and *nimB*-complemented R20291 strains, cultures were grown overnight in BHI with thiamphenicol (15 µg/mL). MICs were performed on BHI agar containing MTZ (0.25–8 µg/mL), hemin (5 µg/mL), ART (128 µg/mL), anhydrotetracycline (ATc; 0.032 µg/mL), and thiamphenicol (15 µg/mL). Control plates lacked ATc and/or hemin.

### Serial passage assay for resistance emergence

A 0.5% overnight culture of strain 17/27 was grown in subinhibitory concentrations of MTZ (1/8× MIC) or the MTZ-ART combination (1/8× MIC each), with both conditions supplemented with hemin (5  µg/mL), and incubated for 48 h. After each 48-h period, MICs were determined for cultures from both conditions. Subsequently, 0.5% of each culture was transferred to fresh media containing the same subinhibitory concentrations, and this process was repeated for eight passages. If no change in MICs was observed, the subinhibitory concentrations were increased to 1/4× MIC, and serial passages were continued for an additional 8 passages, resulting in a total of 16 passages.

### Cytotoxicity evaluation

HEK293T cells (passage numbers 15–25) were grown in Dulbecco’s modified Eagle’s medium (with 4.5 g/L glucose and l-glutamine; Wisent #319-015-CL) supplemented with 2 mM l-glutamine, 1 mM sodium pyruvate, and 10% fetal bovine serum at 37°C in 5% CO_2_. For experiments, cells were seeded at 75,000 cells per well in 24-well plates. After 18 hours, cells were treated with combinations of DMSO, MTZ (0.5 µg/mL), and ART (128 µg/mL). Cytotoxicity was assessed at 5 and 24 h using a Trypan Blue exclusion assay. Briefly, cells were dissociated using a p1000 pipettor by pipetting up and down 10 times, then 20 µL of a 1:1 dilution of cells:0.4% Trypan Blue was prepared. Total and live cell counts were recorded by a BIORAD TC20 Automated Cell Counter. The percent of live cells was used as an indication of cell survival. A combination of cycloheximide at 10 μg/mL and staurosporine at 1 μM (CHX + STS) was used as a positive control.

### Promoter activity

P*nimB*^G^::mCherryOpt or P*nimB*^T^::mCherryOpt constructs were made similar to the previously described method (9). Briefly, a 504 bp upstream region containing the *nimB* promoter was amplified using primers P*nim*Only_NheI_1728_F2 and P*nim*Only_SacI_1728_R2 (Table S3) and cloned into the NheI and SacI sites of the pDSW1728 vector (42). The cloning was used to generate the P*nimB*^G^::mCherryOpt and P*nimB*^T^::mCherryOpt constructs from strains R20291 and CD196, respectively. The constructs and EVs were conjugated into R20291 using *E. coli* SD46. For promoter activity assay, the constructs bearing P*nimB*^G^::mCherryOpt, P*nimB*^T^::mCherryOpt, or EV were grown to OD_600_ ~ 0.3 using 5% overnight cultures. The mid-exponential phase cultures were then treated with MTZ (2 µg/mL) plus hemin (5 µg/mL) with or without ART (128 µg/mL) and incubated anaerobically for 1 h. Samples were kept in the refrigerator overnight to allow mCherry fluorophore maturation. Fluorescence was measured in a BioTek Synergy reader at an excitation of 554 nm and emission of 610 nm, along with OD_600_ (42). Fluorescence of each sample was normalized to its OD_600_.

### FICI

Fractional inhibitory concentration (FIC) was determined using the checkerboard assay in a 96-well plate, following a previously described method with modifications (43). Briefly, a 20 µg/mL hemin solution was prepared in 12 mL of BHI broth, with 100 µL added to all wells. Three working solutions in BHI were prepared: the first containing MTZ (32 µg/mL) with hemin (20 µg/mL), the second containing MTZ (64 µg/mL) with hemin (20 µg/mL), and the third containing ART (2,048 µg/mL). In row A, columns 1–11, 100 µL of the first working solution was added. Then, 100 µL of the second working solution was added to row A of column 12. The contents were serially diluted from rows A to G (second-to-last row). Next, 100 µL of the third working solution (ART 2,048 µg/mL) was added to column 12, rows A to H, and serially diluted right to left to column 2. The plate was covered with aluminum foil and pre-reduced for 3 h before inoculation. After reduction, 100 µL of 0.5% overnight cultures (R20291 and 17/27) were inoculated into each well and incubated for 24 h. The FIC of MTZ and ART was calculated as the MIC of each compound (MTZ or ART) in combination divided by the MIC of each alone. The FICI was the sum of the FICs of the two compounds (FICI = FIC_MTZ_ + FIC_ART_). The result was interpreted as follows: FICI ≤ 0.5: Synergy; FICI > 4: Antagonism; FICI > 0.5–4: No interaction.

### Reverse transcription quantitative real-time PCR

A 5% overnight R20291 culture was grown in BHI broth to an OD_600_ of 0.2–0.3. Cultures were treated with MTZ (2 µg/mL), MTZ (2 µg/mL) plus hemin (5 µg/mL), MTZ (2 µg/mL) plus hemin (5 µg/mL) plus ART (128 µg/mL), ART (128 µg/mL), or DMSO control. After 30 min of anaerobic incubation, cells were collected by centrifugation at 4,000 rpm for 5 min and resuspended in 1 mL RNAprotect Bacterial Reagent (Qiagen, Cat. No. 76506) to preserve RNA. Pellets were stored until RNA extraction. Cell pellets were resuspended in RNA extraction buffer and combined with 300 mg of 0.1 mm disruptor beads (Electron Microscopy Sciences). Cells were lysed using an Omni Bead Ruptor at a speed of 6.6 for 45 s. Lysates were centrifuged at 10,000 × *g* for 3 min, and the supernatants were transferred to fresh tubes. Total RNA was extracted using the HiPure Total RNA Mini Kit (GeneBio Systems, Canada; Cat. No. R401102) and treated with TURBO DNase (Thermo Fisher; Cat. No. AM1907) to remove genomic DNA. cDNA synthesis was performed with qScript cDNA SuperMix (Quantabio, Cat. No. 95048-100), and qPCR was conducted with PerfeCTa SYBR Green SuperMix (Quantabio, Cat. No. 95074-012) on a QuantStudio 6 Flex Real-Time PCR System. Gene expression was normalized to 16S rRNA and calculated using the ΔΔCT method. Primers for qPCR are listed in Table S3.

### Quantification of intracellular hemin levels

A 5% overnight culture of *C. difficile* R20291 was grown to mid-exponential phase (OD_600_ of 0.2–0.3) and divided into four 20 mL aliquots. Each aliquot was treated with one of the following: DMSO (vehicle control), ART (128 µg/mL), hemin (5 µg/mL), or a combination of ART and hemin. Cultures were incubated anaerobically for 24 h, protected from light by covering with aluminum foil. Following incubation, cells were harvested by centrifugation at 5,000 × *g* for 5 min. The resulting pellets were washed three times with PBS to remove extracellular hemin, with centrifugation at 4,000 × *g* for 2 min between washes. The washed pellets were then resuspended in 500 µL of nuclease-free water. To each suspension, 300 mg of 0.1 mm disruptor beads (Electron Microscopy Sciences) were added, and cells were lysed using Omni Bead Ruptor at a speed of 6.6 for 45 s. Lysates were centrifuged at 10,000 × *g* for 3 min, and the supernatants were transferred to fresh tubes. Intracellular hemin levels were quantified using the Hemin Colorimetric Assay Kit (Cat. No. AgK-354, Lifeasible, New York, USA) according to the manufacturer’s instructions. Total protein concentrations were determined using the Pierce BCA Protein Assay Kit (Thermo Fisher Scientific). Hemin concentrations were normalized to total protein content.

## SUPPLEMENTAL MATERIAL

Supplemental Material spectrum.01787-25-s0001.docx Figures S1 to S6; Tables S1 to S3.

ASM does not own the copyrights to Supplemental Material that may be linked to, or accessed through, an article. The authors have granted ASM a non-exclusive, world-wide license to publish the Supplemental Material files. Please contact the corresponding author directly for reuse.
