# Supplementary material for: Reversion of heme-dependent metronidazole resistance in Clostridioides difficile
Source: Microbiol Spectr. 2025 Nov 14;14(1):e01787-25. doi: 10.1128/spectrum.01787-25 (PMC12772251; doi:10.1128/spectrum.01787-25)
Supplement: Supplemental Material — Figures S1 to S6; Tables S1 to S3. [file spectrum.01787-25-s0001.docx]

**Supplementary Information**

**
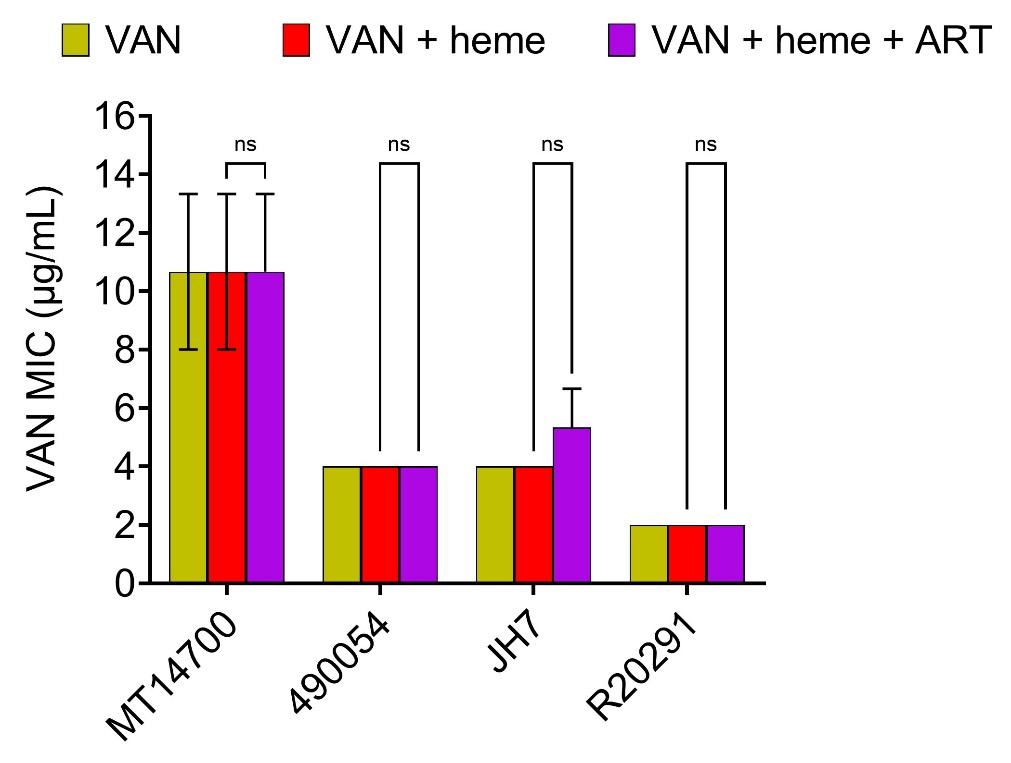
**

**Fig. S1**. ART did not restore vancomycin (VAN) susceptibility in resistant strains (MT14700, 490054, and JH7). R20291, a vancomycin-susceptible strain, serves as the control. Data represent the mean ± SEM from three biological replicates. ns: P > 0.05 (Two-way ANOVA with Holm-Šídák multiple comparisons test).

**
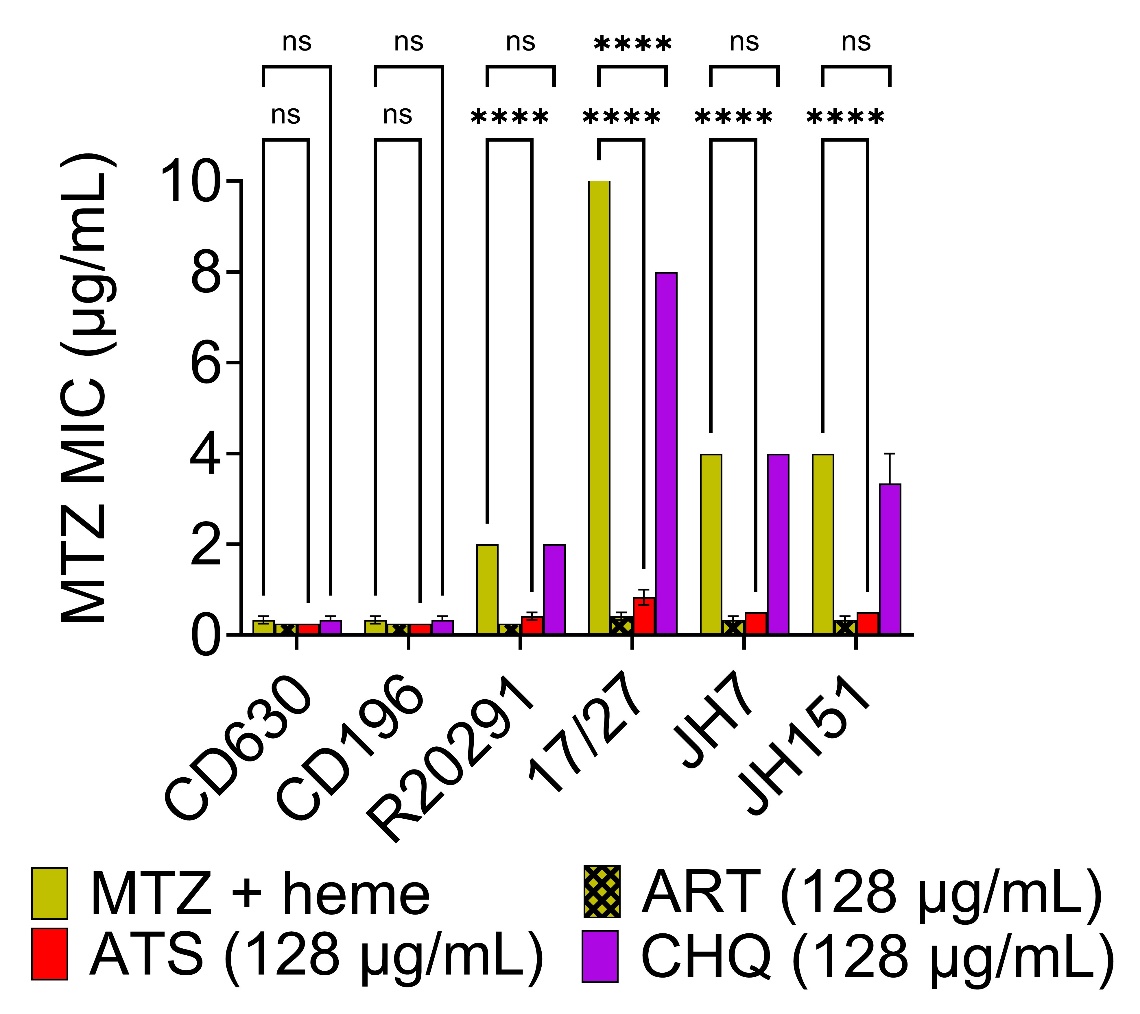
**

**Fig. S2.** Assessment of artesunate (ATS), an artemisinin (ART) analog, showed that ATS similarly reversed MTZ-heme–dependent non-susceptibility in *C. difficile* to or below the non-susceptible cutoff (1 µg/mL), whereas chloroquine (CHQ), a non-artemisinin drug, was ineffective. Data represent the mean ± SEM from three biological replicates. ns: P > 0.05; ****: P ≤ 0.0001 (Two-way ANOVA with Tukey's multiple comparisons test).

**
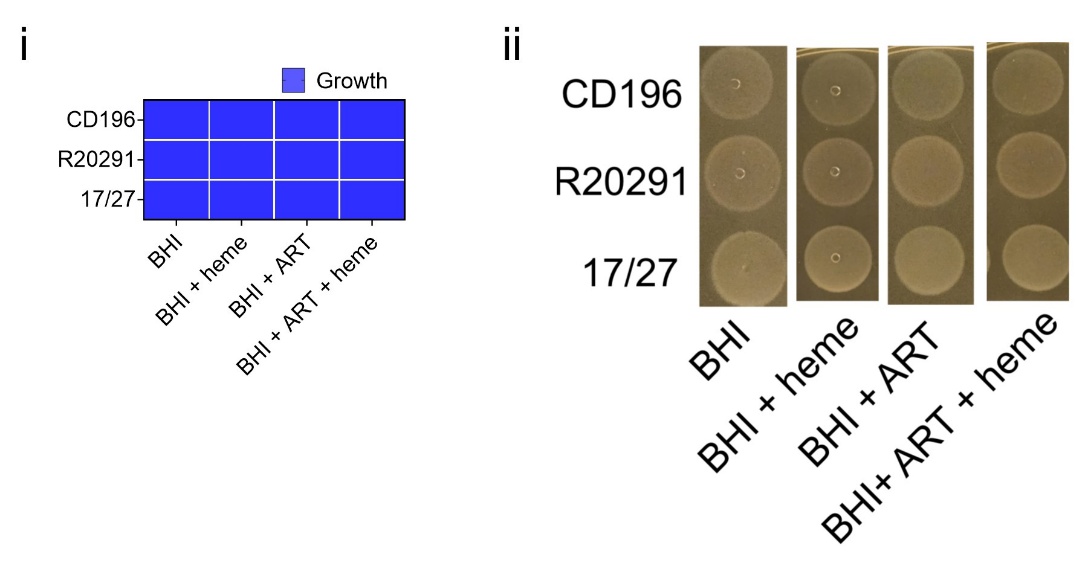
**

**Fig. S3. Growth of *C. difficile* in the presence of heme and ART.** The growth of *C. difficile* was not affected by ART (128 µg/mL) alone or in combination with heme. Growth is indicated by blue color.

**
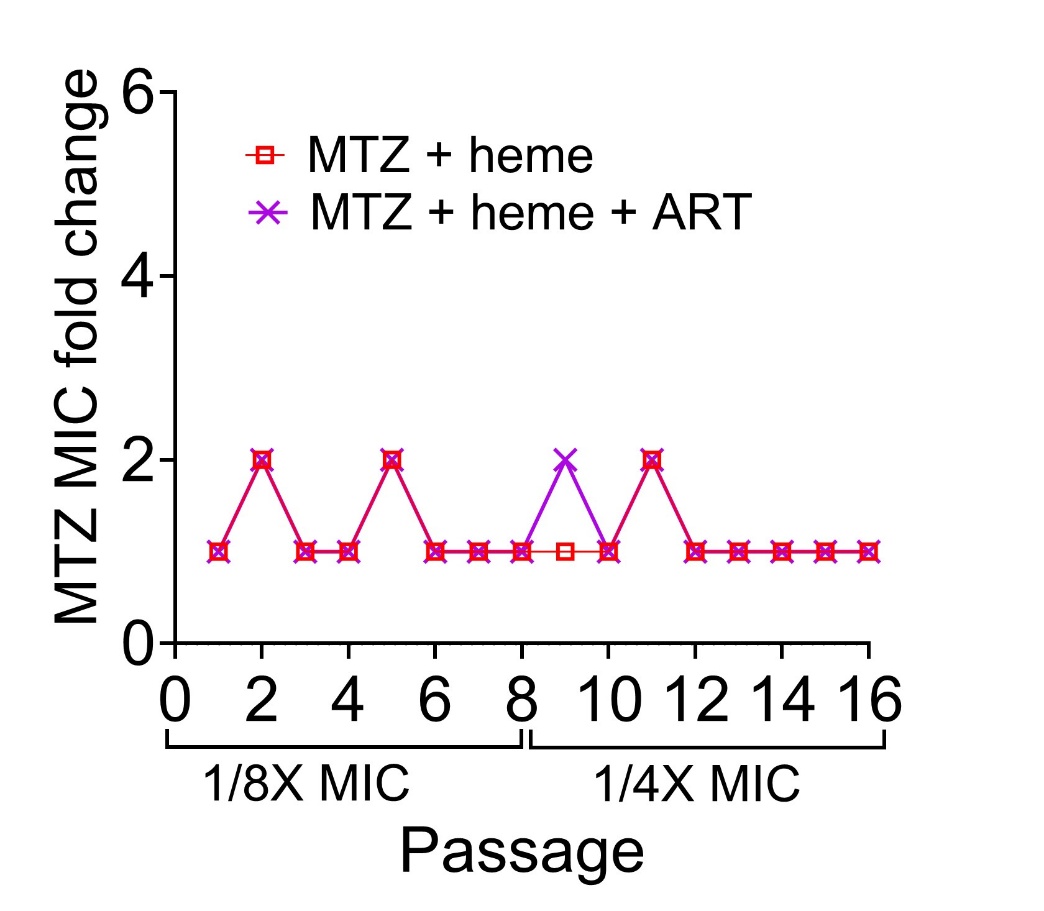
**

**Fig. S4.** Serial passages of *C. difficile* strain 17/27 under subinhibitory concentrations (1/8× MIC and 1/4× MIC) of the MTZ-ART combination in the presence of heme showed no emergence of MTZ resistance over 16 passages.

**
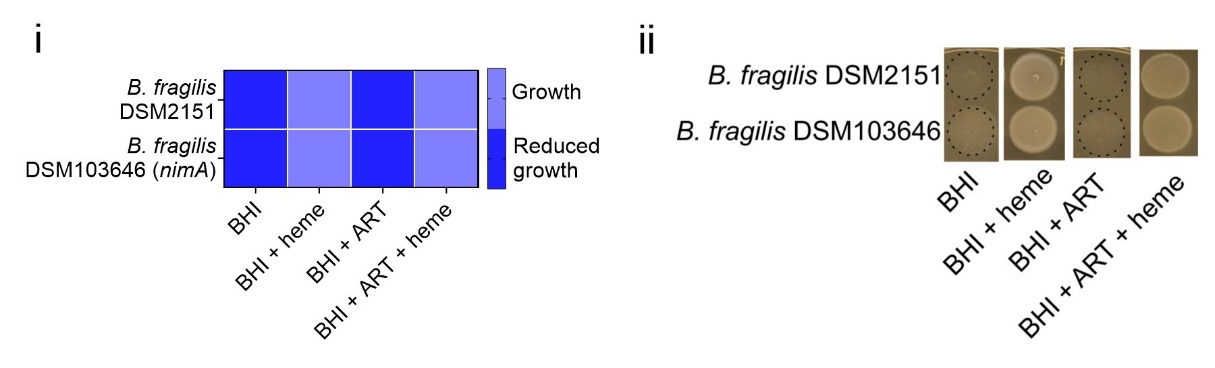
**

**Fig. S5. Growth of *B. fragilis* in the presence of ART.** *B. fragilis* showed reduced growth in BHI alone or with ART (128 µg/mL) without heme. In contrast, robust growth was observed when heme was added to either BHI or ART-containing plates. Growth is indicated in blue, while reduced growth is shown in light blue.

**Table S1:** List of drugs used for screening

| Drug | Catalog number | Supplier | Reference |
| --- | --- | --- | --- |
| 2-Aminopyridine | A77997-5G | Sigma-Aldrich | ^1^ |
| Artemisinin | TRC-A777500-500MG | Toronto Research Chemicals | ^2^ |
| Chloroquine | TRC-C380318-100MG | Toronto Research Chemicals | ^3^ |
| Fluconazole | 37877 | CHEM-IMPEX INT'L. INC. | ^4^ |
| Geldanamycin | 24174 | CHEM-IMPEX INT'L. INC. | ^5^ |
| Imidazole | I202-25G | Sigma | ^6^ |
| Metformin | TRC-M258815-1G | Toronto Research Chemicals | ^7^ |
| Quinoline | 241571-5G | Sigma-Aldrich | ^8^ |
| Ritonavir | SML0491-10MG | Sigma-Aldrich | ^9^ |
| Ketoconazole | TRC-K186000-500MG | Toronto Research Chemicals | ^10^ |
| Quinacrine Dihydrochloride | TRC-Q550610-500MG | Toronto Research Chemicals | ^11^ |
| Deferoxamine mesylate | 14595-500 | Cayman Chemical Co. | ^12^ |
| Miconazole Nitrate | 15420-1 | Cayman Chemical Co. | ^13^ |

**Table S2**: Complete list of clinical *C. difficile* isolates with their MTZ susceptibility profiles, with or without ART supplementation

| Strain | MTZ_R1 | MTZ_R2 | MTZ+heme  _R1 | MTZ+heme  _R2 | MTZ+heme  +ART_R1 | MTZ+heme  +ART_R2 | Ribotype |
| --- | --- | --- | --- | --- | --- | --- | --- |
| 17/27 | 0.5 | 0.25 | 8 | 8 | 0.5 | 0.5 | 001 |
| 7/34 | 0.25 | 0.5 | 8 | 8 | 0.5 | 0.5 | 001 |
| IT1001 | 0.5 | 0.5 | 8 | 8 | 0.5 | 0.5 | 010 |
| IT1002 | 0.5 | 0.5 | 8 | 4 | 0.5 | 0.5 | 010 |
| NR49278 | 0.25 | 0.25 | 4 | 1 | 0.25 | 0.25 | 027 |
| NR49279 | 0.25 | 0.25 | 4 | 2 | 0.25 | 0.25 | 027 |
| NR49284 | 0.25 | 0.5 | 4 | 4 | 0.25 | 0.25 | 027 |
| NR13427 | 0.5 | 0.25 | 4 | 2 | 0.5 | 0.25 |  |
| JH151 | 0.25 | 0.25 | 4 | 4 | 0.25 | 0.25 | 027 |
| NR49289 | 0.25 | 0.25 | 2 | 2 | 0.25 | 0.25 | 027 |
| NR13428 | 0.5 | 0.25 | 2 | 1 | 0.5 | 0.25 |  |
| NR49286 | 0.25 | 0.25 | 2 | 1 | 0.25 | 0.25 | 027 |
| NR49281 | 0.25 | 0.25 | 2 | 2 | 0.25 | 0.25 | 027 |
| NR49288 | 0.25 | 0.25 | 2 | 1 | 0.25 | 0.25 | 027 |
| NR49287 | 0.25 | 0.25 | 2 | 2 | 0.25 | 0.25 | 027 |
| NR32889 | 0.25 | 0.25 | 2 | 2 | 0.25 | 0.25 |  |
| NR49280 | 0.25 | 0.25 | 2 | 1 | 0.25 | 0.25 | 027 |
| JH173 | 0.5 | 0.25 | 2 | 1 | 0.25 | 0.25 | 053-163 |
| JH175 | 0.25 | 0.25 | 2 | 1 | 0.25 | 0.25 | FP310 |
| JH148 | 0.25 | 0.25 | 2 | 1 | 0.25 | 0.25 | 027 |
| JH155 | 0.25 | 0.25 | 2 | 1 | 0.25 | 0.25 | 027 |
| R20291 | 0.25 | 0.25 | 2 | 1 | 0.25 | 0.25 | 027 |
| NR49313 | 0.25 | 0.25 | 2 | 1 | 0.25 | 0.25 | 017 |
| NR32883 | 0.25 | 0.25 | 1 | 1 | 0.25 | 0.25 |  |
| UK1 | 0.25 | 0.25 | 1 | 1 | 0.25 | 0.25 |  |
| JH149 | 0.25 | 0.25 | 1 | 1 | 0.25 | 0.25 | 027 |
| NR49282 | 0.25 | 0.25 | 0.5 | 0.25 | 0.25 | 0.25 | 019 |
| NR49324 | 0.25 | 0.25 | 0.5 | 0.25 | 0.25 | 0.25 | 126 |
| HM746 | 0.25 | 0.25 | 0.5 | 0.25 | 0.25 | 0.25 |  |
| NR49300 | 0.25 | 0.25 | 0.5 | 0.25 | 0.25 | 0.25 | 020 |
| NR49290 | 0.25 | 0.25 | 0.5 | 0.25 | 0.25 | 0.25 | 019 |
| NR49301 | 0.25 | 0.25 | 0.5 | 0.25 | 0.25 | 0.25 | 020 |
| NR49316 | 0.25 | 0.25 | 0.5 | 0.25 | 0.25 | 0.25 |  |
| NR49321 | 0.25 | 0.25 | 0.5 | 0.25 | 0.25 | 0.25 | 106 |
| NR49320 | 0.5 | 0.25 | 0.5 | 0.25 | 0.25 | 0.25 | 106 |
| NR49311 | 0.5 | 0.25 | 0.5 | 0.25 | 0.5 | 0.25 | 78 |
| NR49319 | 0.5 | 0.25 | 0.5 | 0.25 | 0.5 | 0.25 | 106 |
| NR49317 | 0.25 | 0.25 | 0.5 | 0.25 | 0.25 | 0.25 | 024 |
| NR49299 | 0.25 | 0.25 | 0.5 | 0.25 | 0.25 | 0.25 | 020 |
| NR32904 | 0.25 | 0.25 | 0.5 | 0.25 | 0.25 | 0.25 |  |
| NR32903 | 0.5 | 0.25 | 0.5 | 0.25 | 0.25 | 0.25 |  |
| CD630 | 0.5 | 0.25 | 0.5 | 0.25 | 0.5 | 0.25 | 012 |
| CD196 | 0.5 | 0.25 | 0.5 | 0.5 | 0.25 | 0.25 | 027 |
| ATCC700057 | 0.25 | 0.25 | 0.5 | 0.25 | 0.5 | 0.25 | 038 |
| NR49319 | 0.5 | 0.25 | 0.5 | 0.25 | 0.25 | 0.25 | 106 |
| NR49304 | 0.5 | 0.25 | 0.5 | 0.25 | 0.5 | 0.25 | 020 |
| DSM10500 | 0.25 | 0.25 | 0.5 | 0.25 | 0.25 | 0.25 |  |
| NR49296 | 0.25 | 0.25 | 0.25 | 0.25 | 0.25 | 0.25 | 014 |
| NR49303 | 0.25 | 0.25 | 0.25 | 0.25 | 0.25 | 0.25 | 020 |
| NR49307 | 0.25 | 0.25 | 0.25 | 0.25 | 0.25 | 0.25 | 002 |
| NR49309 | 0.25 | 0.25 | 0.25 | 0.25 | 0.25 | 0.25 | 002 |
| NR49308 | 0.25 | 0.25 | 0.25 | 0.25 | 0.25 | 0.25 | 002 |
| HM747 | 0.25 | 0.25 | 0.25 | 0.25 | 0.25 | 0.25 |  |
| HM88 | 0.25 | 0.25 | 0.25 | 0.25 | 0.25 | 0.25 |  |
| NR49326 | 0.25 | 0.25 | 0.25 | 0.25 | 0.25 | 0.25 | 054 |
| NR49325 | 0.25 | 0.25 | 0.25 | 0.25 | 0.25 | 0.25 | 054 |
| NR49328 | 0.25 | 0.25 | 0.25 | 0.25 | 0.25 | 0.25 | 014 |
| NR49327 | 0.25 | 0.25 | 0.25 | 0.25 | 0.25 | 0.25 | 054 |
| NR49329 | 0.25 | 0.25 | 0.25 | 0.25 | 0.25 | 0.25 | 014 |
| NR49323 | 0.25 | 0.25 | 0.25 | 0.25 | 0.25 | 0.25 | 018 |
| NR49314 | 0.25 | 0.25 | 0.25 | 0.25 | 0.25 | 0.25 | 047 |
| NR49305 | 0.25 | 0.25 | 0.25 | 0.25 | 0.25 | 0.25 | 002 |
| NR49304 | 0.25 | 0.25 | 0.25 | 0.25 | 0.25 | 0.25 | 020 |
| NR49297 | 0.25 | 0.25 | 0.25 | 0.25 | 0.25 | 0.25 | 014 |
| NR49306 | 0.25 | 0.25 | 0.25 | 0.25 | 0.25 | 0.25 | 002 |
| NR49295 | 0.25 | 0.25 | 0.25 | 0.25 | 0.25 | 0.25 | 014 |
| NR49294 | 0.25 | 0.25 | 0.25 | 0.25 | 0.25 | 0.25 | 014 |
| NR49293 | 0.25 | 0.25 | 0.25 | 0.25 | 0.25 | 0.25 | 001-072 |
| NR49291 | 0.25 | 0.25 | 0.25 | 0.25 | 0.5 | 0.25 | 027 |
| NR49292 | 0.25 | 0.25 | 0.25 | 0.25 | 0.25 | 0.25 | 001-072 |
| NR49285 | 0.25 | 0.5 | 0.25 | 0.5 | 0.25 | 0.25 | 027 |
| NR49283 | 0.25 | 0.25 | 0.25 | 0.25 | 0.25 | 0.25 | 027 |
| JH166 | 0.25 | 0.25 | 0.25 | 0.25 | 0.25 | 0.25 | 002 |
| NR49310 | 0.25 | 0.25 | 0.25 | 0.25 | 0.25 | 0.25 | 078 |
| NR49318 | 0.25 | 0.25 | 0.25 | 0.25 | 0.25 | 0.25 | 106 |
| NR49298 | 0.25 | 0.25 | 0.25 | 0.25 | 0.25 | 0.25 | 020 |
| NR49315 | 0.25 | 0.25 | 0.25 | 0.25 | 0.25 | 0.25 | 003 |
| NR49302 | 0.25 | 0.25 | 0.25 | 0.25 | 0.25 | 0.25 | 020 |
| NR49312 | 0.25 | 0.25 | 0.25 | 0.25 | 0.25 | 0.25 | 017 |

R_1: biological replicate 1, R_2: biological replicate 2

**Table S3:** List of primers

| **Primers** | **Sequence 5′ – 3′** |
| --- | --- |
| *dacF*_qpcr_F | TGCTTGTGTTGCTATGGCTG |
| *dacF*_qpcr_R | AGTGGTTTGCAACTGGAAGTC |
| *groES*_qpcr_F | ACCAGGAGCAGCTAAAGAGC |
| *groES*_qpcr_R | CCTTATCTCCCACTGTCAATTCCA |
| *dnak*_qpcr_F | ATCCCCTGCTAAAACTCCAGC |
| *dnak*_qpcr_R | ACCAGCAGTACAAGAAGCTGTT |
| 16S rRNA_qpcr_F | CTGGGAGACTTGAGTGCAGG |
| 16S rRNA_qpcr_R | GCCTCAGCGTCAGTTACAGT |
| *hatT*_qpcr_KD_F | GTGGTGTTTACCTTGAATCATAATACT |
| *hatT*_qpcr_KD_R | CAGAAGCGTATGTTAATGTGTATATGGT |
| *hsmA*_qpcr_KD_F | ATGAATTATAAATTAATACTTGCAAT |
| *hsmA*_qpcr_KD_R | CCTGTAATACTATGAAGGTTTTGTGATA |
| PnimOnly_NheI_1728_F2 | TAAGCAGCTAGCCACTTACTGTTAAGCCACCT |
| PnimOnly_SacI_1728_R2 | TGCTTAGAGCTCAATATCATCCTTTCTAATTTTGATGCT |

**References**

1. Kang S, Tang W, Li H, *et al.* Nitric oxide synthase inhibitors that interact with both heme propionate and tetrahydrobiopterin show high isoform selectivity. *J Med Chem* 2014; **57**: 4382–96.

2. Quadros HC, Silva MCB, Moreira DRM. The Role of the Iron Protoporphyrins Heme and Hematin in the Antimalarial Activity of Endoperoxide Drugs. *Pharmaceuticals (Basel)* 2022; **15**: 60.

3. Gorka AP, de Dios A, Roepe PD. Quinoline drug-heme interactions and implications for antimalarial cytostatic versus cytocidal activities. *J Med Chem* 2013; **56**: 5231–46.

4. Hunsaker EW, Franz KJ. Candida albicans reprioritizes metal handling during fluconazole stress. *Metallomics* 2019; **11**: 2020–32.

5. Billecke SS, Bender AT, Kanelakis KC, *et al.* hsp90 is required for heme binding and activation of apo-neuronal nitric-oxide synthase: geldanamycin-mediated oxidant generation is unrelated to any action of hsp90. *J Biol Chem* 2002; **277**: 20504–9.

6. Kokhan O, Shinkarev VP, Wraight CA. Binding of Imidazole to the Heme of Cytochrome c1 and Inhibition of the bc1 Complex from Rhodobacter sphaeroides. *J Biol Chem* 2010; **285**: 22513–21.

7. Li X, Wang X, Snyder MP. Metformin Affects Heme Function as a Possible Mechanism of Action. *G3 (Bethesda)* 2019; **9**: 513–22.

8. Sullivan DJ. Quinolines block every step of malaria heme crystal growth. *Proc Natl Acad Sci U S A* 2017; **114**: 7483–5.

9. Sevrioukova IF, Poulos TL. Structure and mechanism of the complex between cytochrome P4503A4 and ritonavir. *Proc Natl Acad Sci U S A* 2010; **107**: 18422–7.

10. Kartsev V, Geronikaki A, Zubenko A, *et al.* Synthesis and Antimicrobial Activity of New Heteroaryl(aryl) Thiazole Derivatives Molecular Docking Studies. *Antibiotics (Basel)* 2022; **11**: 1337.

11. Choi CYH, Schneider EL, Kim JM, *et al.* Interference with Heme Binding to Histidine-Rich Protein-2 as an Antimalarial Strategy. *Chemistry & Biology* 2002; **9**: 881–9.

12. Premawardhena A, Wanasinghe S, Perera C, *et al.* Deferoxamine, deferasirox, and deferiprone triple iron chelator combination therapy for transfusion-dependent β-thalassaemia with very high iron overload: a randomised clinical trial. *Lancet Reg Health Southeast Asia* 2024; **30**: 100495.

13. Nobre LS, Todorovic S, Tavares AFN, *et al.* Binding of azole antibiotics to Staphylococcus aureus flavohemoglobin increases intracellular oxidative stress. *J Bacteriol* 2010; **192**: 1527–33.
